# Comparative Analysis of Concurrent (CC), Mixed Flow (MX), and Combined Spray Drying Configurations on the Physicochemical Characteristics of Satsuma Mandarin (*Citrus unshiu*) Juice Powders

**DOI:** 10.3390/foods12183514

**Published:** 2023-09-21

**Authors:** Javier Cruz-Padilla, Vondel Reyes, George Cavender, Arranee Chotiko, James Gratzek, Kevin Mis Solval

**Affiliations:** 1 Department of Food Science and Technology, University of Georgia, Griffin, GA 30223, USA; javier.cruz@uga.edu (J.C.-P.); vondelr@uga.edu (V.R.); james.gratzek@uga.edu (J.G.); 2 Department of Food, Nutrition, and Packaging Sciences, Clemson University, Clemson, SC 29634, USA; gcavend@clemson.edu; 3 Division of Food Science and Technology Management, Faculty of Science and Technology, Rajamangala University of Technology Thanyaburi, Bangkok 12110, Thailand; arranee_c@rmutt.ac.th

**Keywords:** satsuma juice powder, spray drying, particle surface area, mixed flow, concurrent

## Abstract

Satsuma mandarins are good sources of vitamin C and can be used as raw materials to produce novel plant-based food ingredients including satsuma mandarin juice powders (SJP). Food powders produced via spray drying often show thermal degradation due to the drying conditions and high drying air temperatures. The aim of this study was to evaluate the effect of using different spray drying configurations, including concurrent (CC), mixed flow (MX), and combined (CC + MX), at two inlet air temperatures (160 and 180 °C) on the physicochemical properties of SJP. Remarkably, SJP produced using the CC spray drying configuration exhibited a higher vitamin C content (3.56–4.01 mg/g) and lower moisture levels (15.18–16.35 g/100 g) than powders produced via MX or CC + MX. The vitamin C content of MX and CC + MX powders ranged from 2.88 to 3.33 mg/g. Meanwhile, all SJP had water activity values below 0.19. Furthermore, MX powders displayed the largest mean particle sizes (D_50_) (8.69–8.83 µm), higher agglomeration, and a rapid dissolution. Despite these differences, all SJP variants exhibited consistent color, surface area, and pore volumes. Notably, powders dried at higher inlet air temperatures (180 °C) showed less vitamin C content and increased thermal damage when compared with powders dried at 160 °C inlet air temperature. This study demonstrated the feasibility of producing high-quality SJP with an extended shelf life. SJP can be used as a novel plant-based ingredient in different food applications.

## 1. Introduction

While satsuma mandarins have been grown in the US for over one hundred years, they are currently seeing a period of rapid growth and have recently become the largest citrus crop in the state of Georgia, with most of the production concentrated in the southern part of the state due to its ideal climate and growing conditions [1]. The harvest season for satsuma mandarins in Georgia typically starts in November and lasts several weeks. As most of the state’s production is intended for the fresh market, the satsuma industry faces several challenges because production often exceeds demand, and the fruits have a shelf life of two–three weeks. This has a downward effect on fruit prices and can lead to product losses [2]. Moreover, by the end of 2023, Georgia is expected to produce over 8.6 million kilograms of satsuma mandarins [3], and the fresh fruit’s short harvesting window and shelf life will continue saturating the US market, further lowering prices and increasing food waste [4]. Hence, it becomes imperative for the American satsuma mandarin industry to explore strategies to add value to its fresh fruit offerings and ensure long-term economic sustainability. In response to this challenge, an opportunity to prolong the shelf life of fresh satsuma mandarins via food innovation has risen. For example, high-quality fruit juice powders can be derived from fresh fruits and used in several plant-based food applications. While some studies have reported the feasibility of producing fruit juice powders via spray drying [5], it is noteworthy that, to the best of the authors’ knowledge, a comprehensive assessment of the effect of spray drying configuration and their interactions with the inlet air temperatures on the quality of the resultant powders have remained unexplored.

Satsuma mandarins are known for their sweet and tangy flavor. As such, they may have a broad spectrum of potential applications in various plant-based foods as natural sweeteners and flavor enhancers. Additionally, they are rich in nutrients and plant-based bioactives, including the vitamins A, C, and E, carotenoids, phenolic compounds, minerals (K, P, Mg), sugars (sucrose, fructose, and glucose), organic acids (citric, malic, and succinic acids), amino acids, and pectin [6,7,8,9,10]. Satsuma juice is high in vitamin C (a water-soluble vitamin and potent antioxidant), beta-cryptoxanthin (an orange–yellow carotenoid and vitamin A precursor), as well as phenolic compounds, including flavonoids and phenolic acids [6,7,9,11].

Producing food powders via spray drying effectively preserves fruit juices’ sensory and nutritional quality [12], and fruit juice powders are high-value products that can be used in several food applications [5]. Spray drying is one of the most cost-effective methods to convert liquid foods, such as fruit juices, into dried and shelf-stable powders [13]. Verma and Singh [14] reported that spray-dried fruit juice powders are easier to handle and transport and have a longer shelf-life than fresh fruits due to their lower moisture content and volume/mass.

The spray drying fruit juices involves three basic steps: atomizing liquid feed, drying the liquid droplets, and recovering the resultant powder [15]. The quality of the final dried powder is affected by several factors, including drying temperatures, liquid feed’s solid concentration, the type and concentration of the drying agent, the liquid feed rate, and the spray drying configuration, especially in fruit juice powders [15]. For example, higher drying temperatures tend to inactivate more significant quantities of heat-sensitive bioactives (compared with lower temperatures); meanwhile, increasing the liquid feed’s solid concentrations and feed rates often results in powders with larger particle sizes and other morphological differences. Even the configuration of the process can affect the product, with certain configurations typically reserved for heat-stable products [13,16,17,18].

Particle morphology can be assessed regarding particle size distribution, particle agglomeration, microstructure, and particle surface area. According to Eijkelboom [19], particle surface properties have a noteworthy impact on stickiness and indirectly influence particle agglomeration. Agglomerated food powders often show excellent functional properties, including higher flowability, reconstitution, bulk density, and mechanical behavior [20]. Moreover, the composition of the liquid feed and the spray drying configuration affect the particle morphology of the resultant spray-dried powders and their functionality. For instance, smooth and spherical particles with few distortions are often associated with free-flowing powders, preferred in dosing, tableting, blending/mixing, and coating [21]. Meanwhile, powder particles with small sizes (<100 µm) and more irregularities (wrinkles and folds) often show inferior flow properties [22]. It has been suggested that agglomerated porous particles (presumably with higher surface areas) often show better flowability, reconstitution behavior, and bulk density [21,22]. It has also been suggested that during the spray drying of liquids, smaller liquid feed droplets show higher drying rates (compared with larger feed droplets) that result in dried particles with high surface areas. However, smaller particles are highly prone to collapsing during drying, which may reduce the surface area [23,24].

The three most common spray drying configurations are concurrent flow (CC), wherein both the sprayed particles and hot air are introduced from the upper part of the drying chamber; counter-current flow (COU), where the sprayed particles and hot air move in opposite directions; and mixed-flow spray drying (MX), which features a combination of both CC and MX configuration [16]. While there is limited literature that simultaneously evaluates various combinations of these conditions, it is essential to acknowledge the potential for interactions among them. In certain instances, leveraging multiple configurations concurrently, such as the CC + MX configuration, is feasible. This involves simultaneously spraying the liquid feed from both the top and bottom of the chamber while keeping the hot air flow consistently in a single direction. Even at the same flow and temperature conditions, the choice of configuration can significantly influence the properties of the resultant powder. For example, our team recently compared the effect of CC and MX on the viability of microencapsulated *Lactobacillus rhamnosus* GG and reported that MX preserved the viability of the probiotic bacteria better than CC [15]. Despite the potential opportunities, studies on producing satsuma mandarin juice powders (SJP) using spray drying remain primarily absent from the scientific literature.

There is considerable information regarding the spray drying of various fruit juices; however, scientific knowledge on the effect of spray drying configuration on the physicochemical quality of the satsuma juice powders remains unknown. This research aims to examine how different spray drying configurations can produce agglomerated particles with higher surface areas. A novel approach emerges wherein CC and MX are harnessed synergistically (CC + MX) to yield an SJP with unique properties.

## 2. Materials and Methods

### 2.1. Material Sources

Fresh satsuma mandarins were obtained from Berry Good farms (Tifton, GA, USA). Maltodextrin (MD, dextrose equivalent = 9–13) was obtained from Now Foods (Bloomingdale, IL, USA). Acetic acid, ascorbic acid, 2,6-Dichloroindophenol, metaphosphoric acid, and sodium bicarbonate were purchased from Sigma Aldrich (St. Louis, MO, USA).

### 2.2. Extraction of Raw Satsuma Mandarin Juice

Satsuma mandarins were manually peeled and refrigerated at 4 °C until needed for juice extraction (total storage time < 24 h), which was performed using a 40 L stainless steel bladder press (0.5 m diameter × 0.58 m height) (Hydro 80Lt, Zambelli, Italy) equipped with a 304 stainless steel jacket and a low-density polyethylene (LDPE) mesh. Municipal tap water was used to inflate the bladder to a pressure of 300 Kpa (43.51 psi), and the expressed raw satsuma mandarin juice (SJ) was collected and stored in a plastic container under refrigeration (4 °C) until needed for analysis and spray drying.

### 2.3. Characterization of SJ

SJ was characterized for total soluble solids (TSS), pH, and color. TSS were quantified using a portable digital handheld refractometer (Atago PAL-1, Cole-Palmer, Vernon Hills, IL, USA), while pH was determined with a benchtop pH meter (Ohaus ST2200-F, Parsippany, NJ, USA). Meanwhile, the color of SJ was characterized with the CIE L * a * b * method using a Lab Scan XE colorimeter (Hunter Associates lab., Inc., Reston, VA, USA) aligned on a top-facing measurement configuration. Chroma and Hue angle values were calculated with the method described by Solval et al. [13]. The total color difference (ΔE) of the powder compared with the fresh juice was estimated using the procedure reported by Jiang et al. [25].

### 2.4. Preparation of Satsuma Mandarin Mixtures

SJ was blended using a magnetic stirring hot plate (Thermo Fisher Scientific, Waltham, MA, USA) and homogenized with a Fisherbrand^TM^ 850 homogenizer (Thermo Fisher Scientific, Waltham, MA, USA) with MD to produce SJ–MD liquid mixtures. MD was added to a 10 g/100 mL SJ ratio. The MD:SJ ratio was selected based on preliminary studies and the TSS of the SJ (~11.8). Our team has previously reported a similar approach [13]. The resultant SJ–MD liquid mixtures were spray dried under different conditions described in Section 2.5.

### 2.5. Spray Drying of SJ–MD

**Figure 1:**
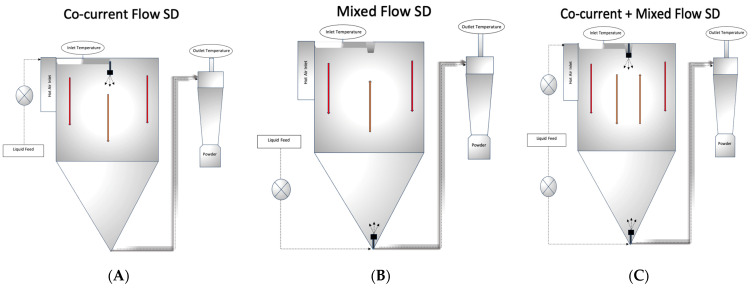
Spray drying configurations used in the study to produce SJP. (**A**) Concurrent spray drying (CC); (**B**) Mixed-flow spray drying (MX); and (**C**) Concurrent + mixed flow spray drying (CC + MX).

The SJ–MD liquid mixtures were spray dried under different conditions, including CC, MX, and CC + MX using an APV Anhydro Electrically heated pilot-scale spray dryer (Anhydro, PSD 52, Soborg, Denmark) with a heat consumption at 350 °C of 12 kW (43.2 MJ) located at the University of Georgia Food Product Innovation and Commercialization Center (UGA FoodPIC) in Griffin, GA. A dual-head peristaltic pump (Masteflex L/S 7523–50, Cole-Palmer, Vernon Hills, IL, USA) was used to feed the product, and a schematic representation of the spray drying configurations are shown in Figure 1. Two inlet air temperatures were used: 160 °C and 180 °C, while feed flow rates were manually adjusted between 1.4 and 1.8 L/hr for all treatments to keep the outlet temperature at 95 °C (±3 °C). To ensure uniformity in flow rate throughout the experiment, the flow rate was divided into two equal portions for the CC + MX configuration. Half of the flow was introduced from the bottom of the drying chamber, while the other half was introduced from the top. This approach was adopted to maintain consistent overall flow rates during the experimental procedures. The resultant spray-dried satsuma juice powders (SJP) were collected from the spray dryer’s cyclonic separator and stored at ambient temperature inside a desiccator until needed for analysis. In total, six SJP were produced: SJP produced under CC at 160 or 180 °C inlet air temperature, SPJ produced under MX at 160 or 180 °C inlet air temperature, and SPJ produced under CC + MX 160 or 180 °C inlet air temperature.

### 2.6. Physicochemical Properties of SJP

#### 2.6.1. Moisture Content and Water Activity

Moisture content was determined using in accordance with the AOAC Official method 934.01 using an Isotemp^®^ Vacuum Oven Model 281A (Thermo Fisher Scientific, Waltham, MA, USA). Water activity was determined according to manufacturer instructions using an Aqualab water activity meter (Model Series 3 TE, Decagon Devices, Inc., Pullman, WA, USA).

#### 2.6.2. Vitamin C

Vitamin C (ascorbic acid) content of SJP was determined using the indophenol method as described by Nielsen [26] using the indicator 2,6-dichloroindophenol. The results were reported as mg of ascorbic acid equivalent/g of powder.

#### 2.6.3. Color

The color of SPJ was determined using a similar approach previously described in Section 2.3. Briefly, a Petri dish was filled with SPJ until the bottom was completely covered with a layer of approximately 10 mm, then the color measurements were collected.

#### 2.6.4. Particle Size Distribution

Powdered samples were assayed for particle size using a Laser Diffraction Particle Size Analyzer (Model PSA 1190, Anton Paar, Graz, Austria) operated in dry sample mode. Size determination was based on the Fraunhofer diffraction and Mie theory with the following parameters: sample read time, 10 s; vibrator duty cycle, 40%; vibrator frequency, 30 Hz; and air pressure, 1300 mBar [27]. Diameter distributions were obtained at 10%, 50%, and 90% cumulative percentile volumes (D_10_, D_50_, and D_90_, respectively). Their results were utilized to calculate the SPAN value to characterize the spread of particles using Equation (1) [20,28].

SPAN = (D_90_ − D_10_)/D_50_ (1)

#### 2.6.5. Dissolution Tests

Dissolution assessment was performed using the procedure outlined by Quek et al. [29], with modifications. Briefly, 50 mg of SJP were placed in a test tube, then one mL of distilled water was added. The resultant mixture was then vortexed using a benchtop mixer Fisher vortex (genie 2, Thermo Fisher Scientific, Waltham, MA, USA) at power level 5 until the sample had been completely reconstituted. The time in seconds required to reach a complete reconstitution of the sample was measured using a digital timer.

#### 2.6.6. Scanning Electron Microscopy (SEM)

The shape and microstructure of the SJP particles were observed with high-resolution SEM micrographs according to the procedure previously reported by our lab [30]. Briefly, samples were coated with a 45-mm film of 80% palladium and 20% gold in an aluminum stub with a Leica EM ACE600 coater (Leica Microsystems, Wetzlar, Germany). Then the micrographs of samples were then captured with a field emission scanning electron microscope (FESEM) (Teneo, Thermo Fisher Scientific, Waltham, MA, USA) at 1000×, 5000× and 10,000× magnifications operating at 5 kV with a resolution of 9.8 nm.

#### 2.6.7. Physisorption Measurements

The surface area analysis and pore volume estimation of the SJP were conducted with N_2_ physisorption at 77.35 K using a NOVATouch LX2 surface area and pore size analyzer (Quantachrome Instruments, Boynton Beach, FL, USA), with nitrogen as the adsorbate gas using the following parameters: a bath delay of 120 s, an adsorption time of 120 s, a desorption time of 20 s, and void volume mode of helium. Prior to each assay, samples were loaded into 9 mm bulbs, and a cleaning process was performed, followed by the degassing of the samples, which was achieved using a vacuum degassing method for 6 h at 100 °C. Surface area was quantified with the Bruneauer–Emmett–Teller (BET) method, and the mesopore distribution was quantified using liquid N_2_ according to the Barrett–Joyner–Halenda (BJH) method [31,32].

### 2.7. Statistical Analysis

All experiments and analytical determinations were performed in triplicate to reduce experimental error. A factorial design was used, where the main effects (SD configurations and inlet temperature) were used to analyze the data in this study. Both of the main effects were assigned and processed as fixed effects. As such, the function of the main effects and interaction satisfies the conditions for an orthogonal set of functions. Post hoc testing, where appropriate, was performed using Tukey’s HSD testing order to identify differences among means. Means testing calculations were performed using statistical software (SAS on Demands for Academics, Cary, NC, USA). In addition, a pairwise Pearson bivariate correlation analysis was utilized to evaluate the correlation between normally distributed linear variables that established linearity between variables using statistical software RStudio statistical software (RStudio, Inc., Boston, MA, USA) separating the correlations between analysis [33,34].

## 3. Results and Discussion

### 3.1. Characterization of the SJ

Fresh Satsuma juice had a TSS of 11.8 ± 0.20 Brix, a pH of 3.66 ± 0.11, and a bright orange color (L* = −62.56; a* = 11.87, b* = 32.71). Approximately 26.75 Kg of SJ and 23.15 Kg of pulp and seeds were obtained from 60 Kg of fresh satsuma mandarins, which equates to a juice extraction yield of 44.65%, which is slightly lower than yields previously reported in other places of the world, where it ranges from 52.9% to 60.1% using various satsuma mandarin cultivars [35]. This is not surprising, as the juice yield among citrus fruits is known to vary depending on the growing conditions, the extraction method/process, fruit maturity, and fruit type. For example, pummelo and lemon typically show a lower juice yield than mandarins and oranges [36]. In general, citrus juices often show TSS values between 9 and 14 Brix, which also varies depending on the cultivar, fruit maturity, and harvesting conditions [35,36]. Similarly, the acidity of the citrus juices is affected by the maturity of the fruit, cultivar, harvesting times, and growing conditions, and further, it has been reported that organic acids such as citric and malic acids in citrus juices are reduced during processing and storage, which often affects fresh fruits’ shelf life and stability [35]. The pH of the juice extracted falls within the range of that extracted from other varieties of satsuma mandarins, which is reported to be between 3.5 and 4.0 [37]. Finally, the extracted SJ had a bright orange color, which is hardly surprising, as according to Tietel et al. [38], the color of satsuma juice is due to carotenoid pigments and further research has reported that the primary carotenoids in satsuma mandarin are β-cryptoxanthin (red), zeaxanthin (orange–red), antheraxanthin (bright yellow), and violaxanthin (orange) [39]. Prior research has documented that satsuma mandarins exhibit the highest carotenoid content compared with other citrus species, leading to their characteristic orange–red hue. Furthermore, variations in the quantities of these carotenoids have been identified across distinct anatomical segments, with the pulp consistently harboring a greater concentration than the peels [40,41].

### 3.2. Physicochemical Properties of SJP

#### 3.2.1. Moisture Content and Water Activity

When liquid foods are spray-dried, they are often exposed to high shear stress within the atomizer, resulting in small liquid droplets exposed to a stream of hot air inside the spray dryer chamber. Subsequently, moisture is quickly evaporated from the droplets by convection [16]. Heat transfer between the drying hot air and liquid feed droplets evaporates moisture [42]. Typically, air inlet temperatures between 150 and 220 °C have been reported in the spray drying of fruit juices [43]. Furthermore, inlet air temperatures between 110 and 190 °C have been used to spray dry citrus juices [42]. Shrestha et al. [44] reported the spray drying of orange juice with maltodextrin using an inlet air temperature of 160 °C with promising results. The researchers noted that the incorporation of maltodextrin increased the resultant powders’ glass transition temperature (Tg). Therefore, our team was interested in evaluating the effects of higher inlet air temperatures (180 °C) on the physicochemical characteristics of the resulting powders. Previous studies have shown that inlet temperature negatively correlates with powders’ final moisture content. In this study, higher inlet temperatures (180 °C) in the MX resulted in powders with significantly (*p* < 0.05) lower moisture content compared with powders produced at inlet air temperatures of 160 °C. However, in the case of CC and CC + MX, the moisture content of the powders was not affected by the inlet air temperature conditions (Table 1). These results may suggest that the drying air temperatures used in this study were not sufficiently high to significantly affect the powders’ moisture content. And while commercially available fruit juice powders are regularly dried at temperatures over 250 °C, such high inlet temperatures may result in the inactivation of heat-sensitive bioactives [43].

Predictably, the moisture content of the powders produced in this study was significantly (*p* < 0.05) affected by the type of SD configuration used in the study. Powders dried at CC conditions showed significantly (*p* < 0.05) lower moisture contents than those produced under MX and CC + MX conditions (Table 1). These results may be due to the liquid droplets’ lower residence times (RT) inside the drying chamber, as higher residence times (RT) often result in drier powder particles [25].

The production of satsuma juice powders with low moisture content has been reported. For instance, when satsuma juice was mixed with 50% maltodextrin and spray-dried, the resultant powders had a moisture content of 8% [6]. However, using 50% maltodextrin may be excessive and impractical. In our study, satsuma juice with 10% maltodextrin was spray-dried, and the resultant powders had higher moisture contents. Despite the higher moisture contents, the a_w_ values for all samples consistently remained below the 0.19 threshold. This low water activity level assures microbial stability, as it resides below the range conducive to the proliferation of most spoilage and pathogenic microorganisms [45].

Water activity is perhaps more important than moisture content, which predicts/indicates a powder’s shelf stability, as it measures the amount of available water in a food product, and all biochemical reactions require some water [29]. In the current study, all the powders obtained showed similar a_w_ values, ranging between 0.14 and 0.19 (Table 1), within the general range for spray-dried fruit juice powders, as they often show a_w_ values below 0.25 [11]. These low values suggest that all the powders produced in this study should be stable during storage, as foods with a_w_ values below 0.3 are microbiologically and enzymatically stable [46,47].

#### 3.2.2. Vitamin C Content

The vitamin C content of SJP ranged from 2.88 to 4.01 mg/g (Table 1). The powders dried at 160 °C inlet air temperature and CC conditions showed significantly (*p* < 0.05) higher vitamin C content than the rest of the powders. Meanwhile, the powders dried at 180 °C inlet air temperature and MX conditions showed the lowest vitamin C contents of all powders.

Vitamin C (aka., L-ascorbic acid) is a water-soluble vitamin naturally found in several fruits and vegetables, including citrus fruits such as satsuma mandarins. Also, it is an essential nutrient for humans; it supports the biosynthesis of collagen, l-carnitine, certain neurotransmitters, and hormones [35,48]. The recommended daily intake of vitamin C varies according to gender, age, pregnancy, or lactation stage, and it ranges from 400 to 2000 mg [49,50], and previous studies have reported a vitamin C content in juice and satsuma segments of 2.81 mg/g and 2.59 mg/g, respectively [51]. Vitamin C has a high redox potential, and it is considered a potent antioxidant due to its ability to donate electrons to free radicals, therefore quenching their reactivity [52]. Although vitamin C is a heat sensitive bioactive, as much as 45% vitamin C retention has been reported after spray drying fruit juices. It has been previously suggested that the levels of vitamin C retention in fruit juice powders after spray drying is strongly influenced by the drying air temperatures and drying agents used [53,54]. In this study, the vitamin C content of the SJ was 5.45 mg/g, higher than a previously reported value. These results suggest that the inlet air temperature and SD configuration were particularly well suited to preserving vitamin C content. This agrees with previous studies that have reported that the air inlet temperature and retention time are the main factors in spray drying that affects the vitamin C content of the spray-dried fruit juice powders; most notably, Estevinho, et al. [53] showed that a reduction in inlet air temperature correlates with increased vitamin C retention [54]. Computational fluid dynamic (CFD) simulation studies of the spray drying of liquid foods have also shown that RTs are affected by SD configurations [55]. As a particle with higher RT would be exposed to the hot air longer, it only follows that the thermal degradation reactions would continue to reduce their vitamin C contents, and one would expect the effect to be more intense at higher drying temperatures. In this study, SJP produced under CC had a higher vitamin C content than powders produced under CC + MX and MX, indicating that powders produced under CC + MX and MX conditions were exposed to hot air in the drying chamber for longer periods than particles dried under CC conditions. It is believed that powders dried via MX experienced extended residence times within the drying chamber, which may have exposed the dried powder particles to elevated temperatures for longer, leading to more significant vitamin C degradation.

#### 3.2.3. Color

The results for the color of SPJ are shown in Table 2. The SJP was whiteish with a pale orange cast, with lightness (L*) values ranging from 10.23 to 12.23; meanwhile, the powders’ greenness/redness (a*) values ranged from 2.90 to 4.12. The lightness of the powders did not appear to be affected by either the SD configurations or the inlet air temperatures. Meanwhile, the redness and blueness/yellowness (b*) values of the SJP were significantly (*p* > 0.05) higher in powders produced via CC + MX. In addition, SJP produced under CC and CC + MX conditions had significantly (*p* < 0.05) higher saturation (Chroma) than the rest of the powders. All SPJ had no significant difference among hue angle and color difference (∆E) values when estimated based on the fresh juice. According to Islam et al. [5], color changes occur after spray drying because of the concentration of solids from the fruit juices and drying agents (maltodextrin, in this case). In this study, SJ was bright orange. It has been reported that adding drying agents can dilute the initial fruit juice color [56]. Previous experiments showed that the carrier agent and inlet temperature increase lightness but reduce the redness and blueness of spray-dried powders [57]. Interestingly, SPJ powders produced via MX showed the lowest yellowness values, possibly due to higher quantities of heat-sensitive bioactives (presumably carotenoids) being inactivated during spray drying [58]. Hue angle is an overall indication of color, and values from 0 to 90 degrees are perceived as reddish, while those from 180 to 270 degrees are perceived as green. Maltodextrin is also known to influence hue angle when used in concentrations between 3% and 10% [59]. While powders with smaller hue angle values may show lower carotenoid content in satsuma juice, as carotenoids are the main cause of its orange color [8], it is important to note that the presence of sugars, pectin, and the added maltodextrin will skew the color towards white, as all of those compounds are essentially white when dry.

#### 3.2.4. Particle Size Distribution

Overall, the results obtained in this study agree with those previously reported in the scientific literature [25]. The spray-dried powders’ mean particle size (D50, µm) ranged from 7.20 to 8.83., with powders dried via MX showing significantly (*p* < 0.05) larger mean and overall particle sizes (D_50_, D_90_) than the powders produced via CC and CC + MX (Table 3). Not surprisingly, the particle size distribution of the powders was not affected by the inlet air temperature. Our team has previously reported that powders dried via MX had higher particle sizes than powders produced via CC [25]. Moreover, higher particle agglomeration (higher span values) were observed in powders produced via MX compared with CC and CC + MX. As the span value is a dimensionless measurement that indicates the particle size spread, so distributions that have a wider range will show higher calculated span values [60]. As with particle size, this suggests that inlet air temperatures does not affect the particle agglomeration (Table 3). Previous studies have reported that larger powder particles can be produced at higher drying temperatures (>250 °C). The size of the resultant spray-dried particles is often determined by the properties of the liquid feed (e.g., solid concentration, viscosity, and acidity), atomization conditions (e.g., shear rates, type of atomizers, etc.), liquid feed flow rate, and SD configurations [43,60]. In addition, high evaporation rates result in small particles [61].

##### Correlation between SD Configuration and Inlet Air Temperature vs. Particle Size Distribution Variables

**Figure 2:**
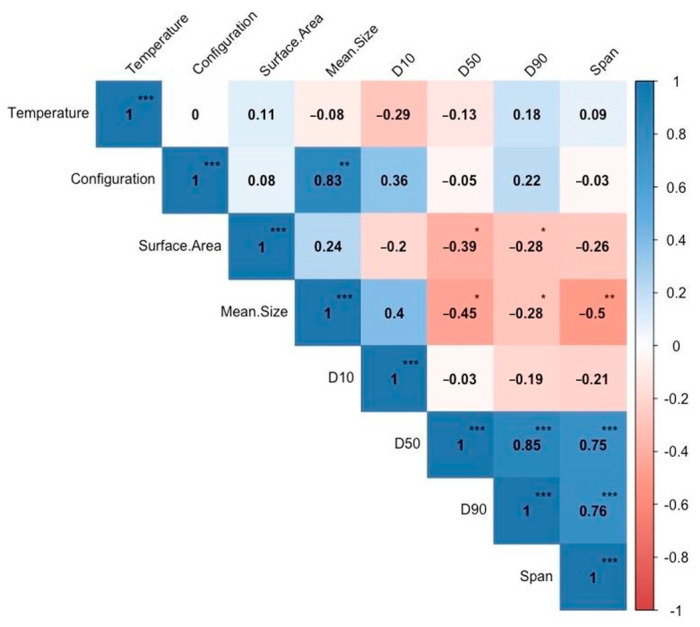
Pairwise Pearson correlation analysis between particle size analysis parameters (D_10_, D_50_, D_90_, and SPAN) and treatment conditions (SD configurations and inlet air temperature). Asterisks represent the *p*-value of the correlations where * *p* < 0.05, ** *p* < 0.01, and *** *p* < 0.001.

The particle size distribution of SJP results were correlated against the levels of main effects (SD configuration and inlet air temperature) using a Pearson correlation approach to better understand the impact of the main effects on the particle sizes of the resultant SJP. A Pearson correlation analysis between random variables is often used to reduce experimental noise [62,63] and, after analysis of the data, it was noted that the SD configuration (CC, MX, and CC + MX) had a highly significant positive correlation (*p* < 0.01) with the mean sizes of the powder particles (Figure 2). However, the results again suggested that the inlet air temperatures did not affect the particle sizes of the spray-dried powders (as indicated by the low correlation values).

#### 3.2.5. Dissolution Test

The dissolution examination assesses the time a powdered substance requires for reconstitution in water [29]. In this study, SPJ produced via MX and MX + CC dissolved significantly (*p* < 0.05) faster than powders produced via CC (Table 4). Remarkably, the inlet air temperature did not affect the dissolution times of the SJP, and no discernible interaction among the main effects was observed. Similar studies have reported the impact of SD configurations on the dissolution properties of fruit juice powders [64].

In the initial phase of the dissolution of powders, water infiltrates the porous matrix of the dried particles in the water-soluble areas (aka. areas with high sugar or other water-soluble compounds) [65]. This may suggest that either powder particles with higher quantities of surface water-soluble compounds were produced via MX and MX + CC compared with CC, or that MX, and MX + CC powders had a more porous structure or both.

#### 3.2.6. Scanning Electron Microscopy

**Figure 3:**
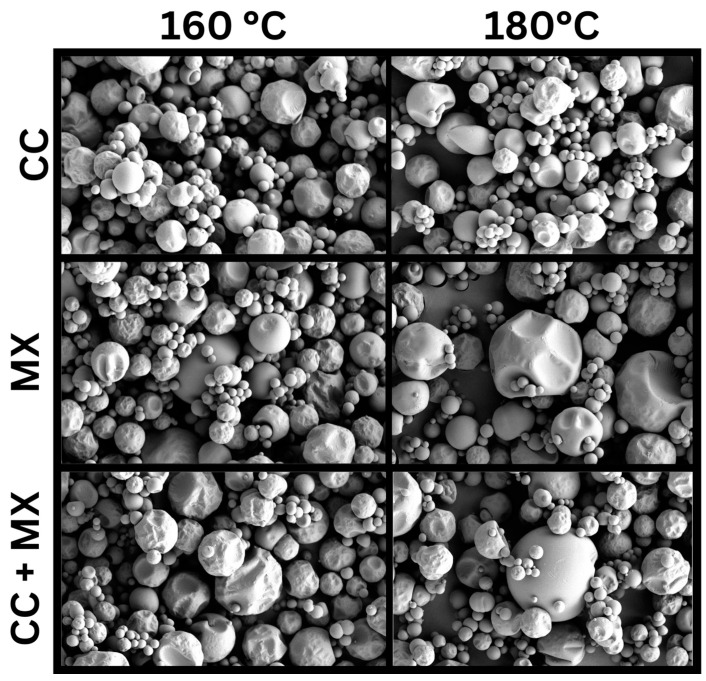
SEM micrographs of SJP powders dried via CC, MX, and CC + MX at 160 and 180 °C inlet air temperatures. CC = concurrent spray drying, MX = mixed-flow spray drying; CC + MX = concurrent and mixed-flow spray drying.

Higher particle agglomeration was observed in the powders produced via MX (Figure 3). Moreover, SJP produced via CC showed fewer signs of damage compared with the powders produced via CC + MX and MX, which may explain some of the previous solubility data.

SEM micrographs allow us to observe damage to the powders’ particles dried under different conditions [66]. The damage caused to powder particles dried under CC and CC + MX conditions was noticeable. In addition, the higher particle agglomeration observed in MX powders confirmed the results previously discussed in Section 3.2.4. The formation of concavities on the particle surface is mainly attributed to modifying particle size during moisture evaporation during spray drying, which causes an inflation and deflation of the particles. Nevertheless, if breakage or cracks on the particles’ surface are present, this suggests a harsh and very fast drying caused by high drying temperatures and RT, and excessive particle coalescence inside the spray dryer [46,67,68]. Also, it is believed that citrus juices have significant amounts of organic acids, such as citric acid, and monosaccharides, such as fructose, which may increase the stickiness of the final powders. It has been reported that the stickiness of spray-dried powder may be predicted by observing particle agglomeration in the SEM micrographs [5].

#### 3.2.7. Particle Surface Area and Total Pore Volume

All SPJ had a similar surface area (m^2^/g) and ranged from 2.88 to 4.93 (Table 4). In addition, SJP dried via CC + MX had a slightly higher pore volume (cc/g) than the other powders. Saifullah et al. [65] stated that higher dissolution times are associated with lower pore volumes. The total pore volume of powders dried via CC + MX ranged from 2.11 to 2.87 × 10^−4^ cc/g. Moreover, the dynamic gravimetric BET method estimates the surface area and pore volume by describing the surface area of solid particles like powders. This method uses isotherms to quantify the amount of gas that builds up on the particle surface. Then, the surface area is calculated by multiplying the total number of molecules in these monolayers by the required space that one molecule requires [31]. Neither the SD configuration nor the inlet air temperature affected the total surface area of the resultant powders. It has been reported that amorphous materials usually show higher surface areas and pore volumes than crystalline materials [69,70]. Powdered particles with higher surface areas may expose higher amounts of bioactives to environmental conditions (e.g., oxygen and light) during processing and storage [71].

### 3.3. Correlation Analysis between SD Configurations and Inlet Air Temperatures vs. Particle Surface Area, Pore Volume, Dissolution, Vitamin C, and Span Values of SJP

**Figure 4:**
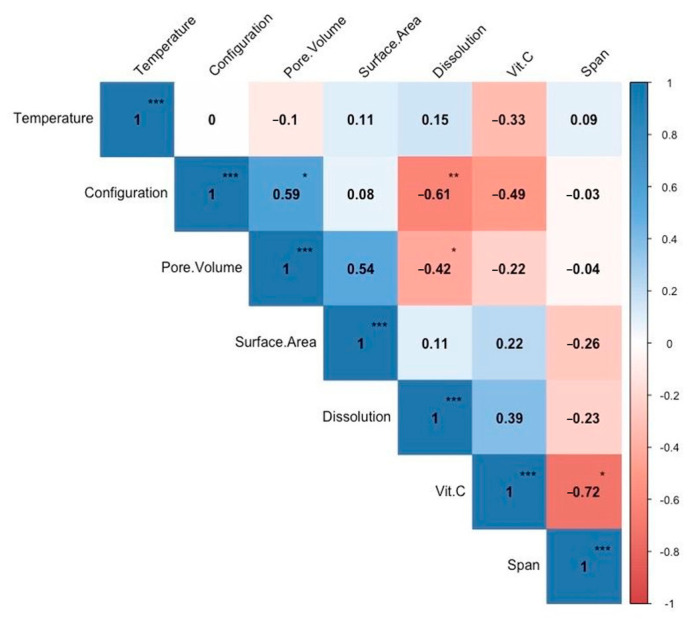
Pairwise Pearson correlation between inlet air temperature and SD configuration vs. several physicochemical properties of SJP. Asterisks represent the *p*-value of the correlations where * *p* < 0.05, ** *p* < 0.01, and *** *p* < 0.001.

A second Pairwise Pearson correlation analysis was conducted in this study (Figure 4). The results suggested that the inlet air temperatures did not significantly correlate with any of the evaluated variables (pore volume, surface area, dissolution, vitamin C content, and span of SJP). However, the SD configuration had a significant (*p* < 0.05) positive correlation with pore volumes of SJP. This suggests that powders with higher pore volumes can be produced via CC + MX. We believe this effect may be possible due to a high particle coalescence and recirculation. During CC + MX, a liquid feed is sprayed from the top and the bottom of the drying chamber of the spray dryer, as observed in Figure 1C. In addition, CC + MX may be more useful in applications that require the production of powders with high pore volumes (e.g., catalysts and enzymes). The results of imperfections in powders with large pores produced via CC + MX can be seen in the SEM micrographs (Figure 3).

Another exciting correlation was between the SD configuration and the dissolution of the SJP. The results suggested a highly significant (*p* < 0.01) negative correlation between those variables. This analysis suggests that SJP produced via CC + MX can dissolve faster in water than SJP produced via CC or MX. Furthermore, SJP with higher surface areas had a significant (*p* < 0.05) positive correlation with the dissolution test (i.e., powders with a higher surface area dissolve faster).

The correlation analysis also revelated a significantly (*p* < 0.05) high negative correlation between vitamin C content and the span value of powders. This suggests that the higher the particle agglomeration, the lower the vitamin C content in the SJP. Even though the inlet air temperatures did not significantly correlate with of the evaluated variables in our study, Solval et al. [13] reported that the vitamin C content of fruit juice powders can be affected by the inlet air temperatures in the CC configuration.

## 4. Conclusions

This study demonstrated that both spray drying configuration and inlet air temperatures affected the physicochemical properties of satsuma mandarin juice powder. The reported findings provide valuable insights into optimizing the production of these powders. Notably, powders produced through CC spray drying exhibited superior properties, including a lower moisture content and higher vitamin C content and dissolution rates than powders produced via MX and CC + MX spray drying configurations. Conversely, powders produced via MX showed higher agglomeration compared with the rest of the powders. Notably, neither the chosen spray drying configuration nor the inlet air temperature significantly influenced the resulting satsuma mandarin juice powders’ surface area or pore volume. Generally, powders dried at 180 °C showed higher thermal degradation than those dried at 160 °C. Therefore, this study highlights the importance of carefully selecting the drying configuration and the inlet air temperature when developing a spray drying process for producing satsuma mandarin juice powders.

Several challenges were encountered during the execution of this study. For example, it was not feasible to use different feed flow rates. In addition, powders were only dried at two inlet air temperatures. Other drying temperatures may have had different effects on the resultant powders. Furthermore, incorporating larger quantities of maltodextrin was observed to enhance the flowability of the resultant product. However, this improvement was accompanied by notable drawbacks, including a discernible loss of color and heightened difficulty in reconstituting the powder in aqueous solutions at later stages. Future research should focus on conducting a comprehensive sensorial analysis to assess the acceptability of the reconstituted satsuma juice. Additionally, a cost analysis of this process warrants investigation to provide a more comprehensive understanding of its economic feasibility. Finally, we recommend investigating the effect of spray drying conditions on the carotenoids and flavor compounds of satsuma mandarin juice powders. This study shows a promising process to produce satsuma mandarin juice powders that can be used as a plant-based ingredient in several food applications.

## Figures and Tables

**Table 1:** Moisture, water activity, and vitamin C content of satsuma juice powders.

SD Configuration	Inlet Temperature (°C)	Moisture Content (g/100 g) ^1^	Water Activity (a_w_) ^1^	Vitamin C (mg/g) ^1^
CC	160	16.35 ± 5.52 ^c^	0.14 ± 0.01 ^a^	4.01 ± 1.31 ^a^
CC	180	15.18 ± 4.74 ^c^	0.14 ± 0.02 ^a^	3.56 ± 2.63 ^a^
MX	160	25.00 ± 0.00 ^a^	0.18 ± 0.02 ^a^	3.18 ± 2.27 ^c^
MX	180	19.72 ± 1.16 ^b^	0.17 ± 0.03 ^a^	2.88 ± 1.31 ^c^
CC + MX	160	25.00 ± 0.00 ^a^	0.19 ± 0.03 ^a^	3.33 ± 1.31 ^b^
CC + MX	180	25.44 ± 0.76 ^a^	0.14 ± 0.01 ^a^	3.33 ± 1.31 ^b^

^1^ Values are means ± standard deviation (SD). ^a,b,c^ Means with the same letter in each column are not significantly different (*p* < 0.05). CC = concurrent spray drying, MX = mixed-flow spray drying; CC + MX = concurrent and mixed-flow spray drying.

**Table 2:** Color of SJP.

Configuration	Inlet Temperature (°C)	L* ^1^	a* ^1^	b* ^1^	Chroma ^1^	Hue Angle ^1^	∆E ^1^
CC	160	10.30 ± 1.17 ^a^	2.97 ± 0.35 ^b^	19.65 ± 1.34 ^b^	19.88 ± 1.30 ^a^	1.42 ± 0.02 ^a^	2.94 ± 1.20 ^a^
CC	180	11.95 ± 1.38 ^a^	2.90 ± 0.50 ^b^	21.58 ± 2.21 ^b^	21.78 ± 2.24 ^a^	1.44 ± 0.02 ^a^	3.10 ± 1.14 ^a^
MX	160	11.34 ± 1.34 ^a^	3.49 ± 0.19 ^b^	18.29 ± 1.61 ^b^	18.62 ± 1.62 ^b^	1.38 ± 0.02 ^a^	2.16 ± 0.22 ^a^
MX	180	10.23 ± 0.90 ^a^	2.94 ± 0.41 ^b^	17.71 ± 0.78 ^b^	17.95 ± 0.78 ^b^	1.41 ± 0.02 ^a^	1.75 ± 0.49 ^a^
CC + MX	160	12.23 ± 0.39 ^a^	4.12 ± 0.34 ^a^	20.43 ± 0.32 ^a^	20.84 ± 0.28 ^a^	1.37 ± 0.02 ^a^	1.14 ± 0.37 ^a^
CC + MX	180	10.68 ± 0.27 ^a^	3.78 ± 0.32 ^a^	22.36 ± 1.89 ^a^	22.68 ± 1.88 ^a^	1.40 ± 0.02 ^a^	3.93 ± 3.41 ^a^

^1^ Values are means ± standard deviation (SD). ^a,b^ Means with the same letter in each column are not significantly different (*p* < 0.05). CC = concurrent spray drying, MX = mixed-flow spray drying; CC + MX = concurrent and mixed-flow spray drying.

**Table 3:** Particle size distribution of SJP.

SD Configuration	Inlet Temperature (°C)	D_10_ [um] ^1^	D_50_ [um] ^1^	D_90_ [um] ^1^	Span ^1^
CC	160	0.25 ± 0.02 ^a^	7.51 ± 0.02 ^b^	8.96 ± 0.05 ^b^	2.32 ± 0.04 ^b^
CC	180	0.20 ± 0.01 ^a^	7.20 ± 0.24 ^b^	9.05 ± 0.19 ^b^	2.61 ± 0.10 ^b^
MX	160	0.23 ± 0.01 ^a^	8.83 ± 0.16 ^a^	26.28 ± 0.27 ^a^	2.95 ± 0.03 ^a^
MX	180	0.22 ± 0.01 ^a^	8.69 ± 0.28 ^a^	26.86 ± 0.86 ^a^	3.06 ± 0.09 ^a^
CC + MX	160	0.36 ± 0.22 ^a^	7.34 ± 0.57 ^b^	9.33 ± 0.59 ^b^	2.57 ± 0.07 ^b^
CC + MX	180	0.25 ± 0.06 ^a^	7.21 ± 0.37 ^b^	16.93 ± 0.60 ^b^	2.32 ± 0.07 ^b^

^1^ Values are means ± standard deviation (SD). ^a,b^ Means with the same letter in each column are not significantly different (*p* < 0.05). CC = concurrent spray drying, MX = mixed-flow spray drying; CC + MX = concurrent and mixed-flow spray drying.

**Table 4:** Particle surface area and total pore volume of SJP.

SD Configuration	Inlet Temperature (°C)	Dissolution (s) ^1^	Surface Area (m^2^/g) ^1^	Total Pore Volume (cc/g) ^1^
CC	160	41.67 ± 7.57 ^a^	4.93 ± 0.17 ^a^	1.79 × 10^−4^ ± 2.91 × 10^−6 a^
CC	180	36.33 ± 4.93 ^a^	4.00 ± 0.61 ^a^	1.20 × 10^−4^ ± 4.66 × 10^−5 a^
MX	160	27.33 ± 2.52 ^b^	2.88 ± 1.41 ^a^	1.34 × 10^−4^ ± 4.19 × 10^−5 a^
MX	180	29.33 ± 1.53 ^b^	4.39 ± 1.90 ^a^	2.22 × 10^−4^ ± 9.00 × 10^−5 a^
CC + MX	160	22.33 ± 4.93 ^b^	4.58 ± 0.97 ^a^	2.87 × 10^−4^ ± 3.90 × 10^−5 a^
CC + MX	180	29.33 ± 5.86 ^b^	4.82 ± 1.72 ^a^	2.11 × 10^−4^ ± 1.94 × 10^−5 a^

^1^ Values are means ± standard deviation (SD). ^a,b^ Means with the same letter in each column are not significantly different (*p* < 0.05). CC = concurrent spray drying, MX = mixed-flow spray drying; CC + MX = concurrent and mixed-flow spray drying.

## Data Availability

Data are contained within the article.
